# Case Report: Bosentan and Sildenafil Exposure in Human Milk - A Contribution From the ConcePTION Project

**DOI:** 10.3389/fphar.2022.881084

**Published:** 2022-06-15

**Authors:** Nina Nauwelaerts, Michael Ceulemans, Neel Deferm, An Eerdekens, Bart Lammens, Yeghig Armoudjian, Kristel Van Calsteren, Karel Allegaert, Loes de Vries, Pieter Annaert, Anne Smits

**Affiliations:** ^1^ Department of Pharmaceutical and Pharmacological Sciences, KU Leuven, Leuven, Belgium; ^2^ L-C&Y, KU Leuven Child & Youth Institute, Leuven, Belgium; ^3^ Teratology Information Service, Netherlands Pharmacovigilance Centre Lareb, ‘s Hertogenbosch, Netherlands; ^4^ Neonatal Intensive Care Unit, University Hospitals Leuven, Leuven, Belgium; ^5^ BioNotus GCV, Niel, Belgium; ^6^ Gynaecology and Obstetrics, UZ Leuven, Leuven, Belgium; ^7^ Department of Development and Regeneration, KU Leuven, Leuven, Belgium; ^8^ Department of Hospital Pharmacy, Erasmus University Medical Center, Rotterdam, Netherlands

**Keywords:** case report, sildenafil, bosentan, pulmonary arterial hypertension, pharmacokinetics, lactation, human milk, breastfeeding

## Abstract

**Introduction:** Quantitative information on disposition of maternal medicines in human milk remains a major knowledge gap. This case report presents the clinical and pharmacokinetic data of a single mother-infant pair exposed to bosentan and sildenafil for the treatment of pulmonary arterial hypertension (PAH) during lactation.

**Case presentation:** A 43-year old mother was treated with sildenafil (20 mg, 3x/day) and bosentan (125 mg, 2x/day) for PAH. Her 21-months old infant received breastfeeding in combination with adequate complementary foods. Milk samples were collected over 24 h, at day 637 and 651 after delivery. The observed average steady-state concentrations of sildenafil (2.84 μg/L) and bosentan (49.0 μg/L) in human milk were low. The Daily Infant Dosage ingested by the nursing infant through human milk was 0.02 μg/kg/day for sildenafil and 0.29 μg/kg/day for bosentan at day 637, and 0.03 μg/kg/day and 0.60 μg/kg/day at day 651. The Relative Infant Dose calculated for an exclusively breastfed infant with an estimated milk intake of 150 ml/kg/day, was 0.06% for sildenafil and 0.24% for bosentan. General health outcome of the infant, reported by the mother, was uneventful until the sampling days.

**Conclusion:** Low medicine concentrations were found in human milk expressed 21 months after delivery after maternal intake of 20 mg sildenafil three times daily and 125 mg bosentan twice daily. General health of the nursing infant until sampling was reported as optimal by the mother.

## Introduction

Sildenafil and bosentan are used to treat pulmonary arterial hypertension (PAH). Sildenafil (molecular weight: 475 g/mol) is an inhibitor of cyclic guanosine monophosphate specific phosphodiesterase type 5. Sildenafil is quickly absorbed (T_max_: 60 min), with a mean absolute oral bioavailability of 41%. Sildenafil is 96% bound to plasma proteins, and metabolized primarily by the enzymes cytochrome P450 (CYP)3A4 and CYP2C9, followed by excretion in feces and urine. The terminal phase half-life is approximately 4 h. Bosentan (molecular weight: 552 g/mol) is an endothelin receptor antagonist, has an absolute bioavailability of 50%, and is highly bound to plasma proteins (>98%). It is metabolized by CYP2C9 and CYP3A4, followed by biliary excretion. The terminal elimination half-life is 5.4 h. The drug-drug interaction between bosentan (125 mg, twice-daily) and sildenafil (80 mg, three times daily) resulted among healthy volunteers in a reduction of 63% in the area under the plasma concentration-time curve (AUC) of sildenafil and an increase of 50% in the AUC of bosentan (Sandoz nv/sa, 2020; Accord Healthcare, 2021).

Pregnancy is contra-indicated for women with PAH (Luo et al., 2020). Consequently, there is little experience with the combined use of sildenafil and bosentan during pregnancy and lactation. Breastfeeding is not recommended during treatment with bosentan, since the extent of transfer into human milk is unknown (Bosentan, 2006). A case report of a 23-year old woman treated with sildenafil and bosentan during pregnancy and postpartum concluded that the infant was nursed with good outcome, although the doses and extent of breastfeeding were not specified (Molelekwa et al., 2005). Another case report of a woman treated with sildenafil during pregnancy and lactation suggested that secretion of sildenafil in human milk is limited (1.64—4.49 μg/L) (Wollein et al., 2016).

Breastfeeding plays an important role in the development and survival of infants, and is associated with positive health effects for mother and nursing infant (Binns et al., 2016). The World Health Organization^1^ recommends exclusive breastfeeding during the first 6 months of life, and continued breastfeeding in combination with safe and adequate complementary foods for up to 2 years and beyond. Although the transfer of most medicines into human milk is expected to be limited, this area has historically only received little attention. Off-label use of medicines during lactation exposes neonates and infants to unknown health risks. Simultaneously, women might decide to discontinue breastfeeding or to avoid initiation of their pharmacotherapy. The case report fits in the Innovative Medicines Initiative ConcePTION^2^, which aims to reduce the uncertainty about the effects of medicines used during pregnancy and lactation.

The aim of this case report was to determine human milk concentrations of bosentan and sildenafil in a lactating mother, to estimate infant exposure, and to provide the reported general health of the nursing infant until the last sampling moment.

This case report was prepared along the CARE Guidelines (Gagnier et al., 2013).

## Case Description: Patient Information and Clinical Setting

This case report includes a single mother-infant pair. The mother was a 43-year old European woman, with a body mass index of 29.1 kg/m^2^ (83 kg, 169 cm). Treatment with sildenafil and bosentan for PAH was initiated more than 6 months after delivery. Approval was obtained from the Ethics Committee Research UZ/KU Leuven (S64702), and the woman provided written informed consent. The mother did not smoke, nor used alcohol or was on a specific diet. She didn’t have renal or hepatic failure. The infant was male, born healthy and term. In the period of human milk sample collections, the infant received breastfeeding (± three times daily) in combination with adequate complementary foods. At that time, he was 21 months old, weighing 11 and 11.2 kg on the first and second sampling day respectively.

## Timeline Section

**FIGURE 1:**
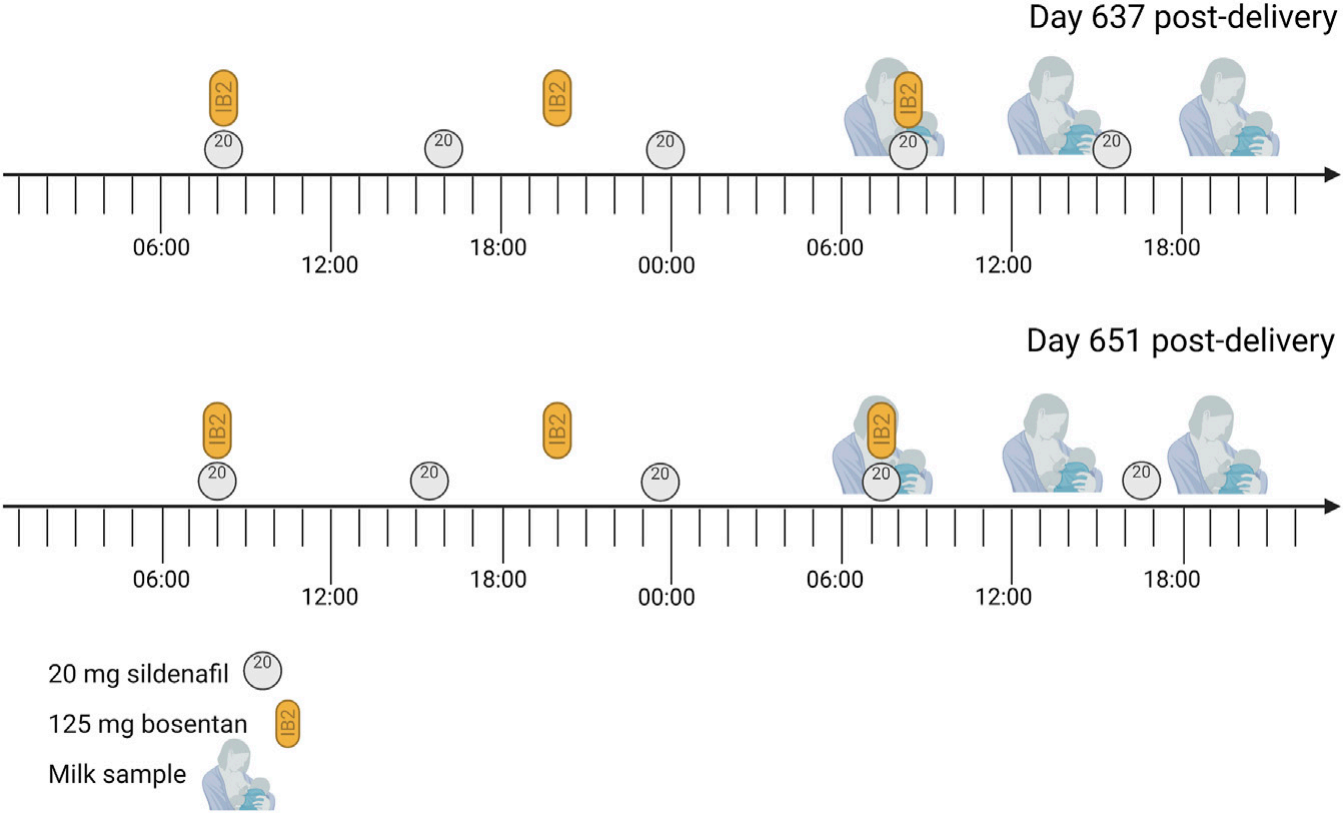
Sampling days The mother was treated with 20 mg sildenafil three times daily and 125 bosentan twice daily for pulmonary arterial hypertension. She also took 25 µg/day Vitamin D3 (1000 IE) each morning and Ibuprofen (400 mg) on day 637 post-delivery at 14:30, but this is not expected to interfere with the studied medicines. Each expression session, the mother collected a sample from the expressed milk.

The sampling days are illustrated in Figure 1.

## Therapeutic Interventions

The mother was treated with Balcoga (sildenafil) 20 mg film-coated tablets (Sandoz nv/sa, Vilvoorde, Belgium) three times daily, and Bosentan Accord 125 mg film-coated tablets (Healthcare B. V., Utrecht, the Netherlands) twice-daily (Table 1). As co-medication, she used Vitamin D3 25 µg (1000 IE, oral spray, Holland and Berrett) each morning and occasionally Ibuprofen (400 mg). Dosing and timing of medicines used are provided in Table 1 and Figure 1.

**TABLE 1:** Overview of the timing and dosing of maternal medicines used.

Days after Delivery	Time (hh:mm)	Name of the medicine	Dose (mg)
636	20:00	Bosentan	125
	23:45	Sildenafil	20
637	8:20	Sildenafil	20
		Bosentan	125
	15:30	Sildenafil	20
650	20:00	Bosentan	125
	23:30	Sildenafil	20
651	7:30	Sildenafil	20
		Bosentan	125
	16:30	Sildenafil	20

## Follow-Up and Outcomes

### Maternal

A case report form was used to collect maternal variables and to record maternal medicine intake during 7 days prior to the two sampling days (Supplementary Figure S1). The mother reported having occasionally experienced nasal congestion and tension headache as possible adverse effects.

### Infant

The case report form, which was completed by the mother at each sampling day, also collected infant variables, including general health outcomes of the infant (i.e. hospital admissions, health problems, adverse effects until sampling days), and medication use 7 days prior to milk sampling. The mother did not report any possible adverse effect, serious health problem or hospitalization of the infant in the period from birth until the last sampling day. The infant did not receive any medicines, except vitamin D suppletion.

### Human Milk Samples

Human milk samples were obtained at steady-state for sildenafil and bosentan, at day 637 (sampling day 1) and 651 (sampling day 2) post-delivery. At both days, human milk was collected over 24 h, starting at the time of medicine intake (Figure 1). The mother used an electric pump to express the total milk volume from both breasts at her home. The milk volume expressed during each session was recorded, and a sample (10 ml) was taken for analysis. The samples were immediately stored in the fridge (4°C) for a maximum of 24 h, and thereafter stored in the freezer (−20°C) for a maximum of 2 months. Long-term storage of the samples was done at −80°C. Transport of the samples was organized with attention for the quality of the samples (e.g. use of dry ice). The samples were analyzed via a liquid chromatography with tandem mass spectrometry (LC-MS/MS) method (Supplementary Data). The human milk samples are summarized in Table 2.

**TABLE 2:** Overview of the timing and volume of collected milk samples along with milk concentrations of sildenafil and bosentan.

Sampling day^a^	Time (hh:mm)	Milk volume (L)	Concentration sildenafil (µg/L)	Concentration bosentan (µg/L)
1	7:30	0.04	2.18	33.0
	13:30	0.03	2.22	54.3
	19:30	0.01	5.65	23.2
2	7:30	0.05	1.43	24.5
	13:25	0.05	2.10	86.1
	19:00	0.04	4.95	30.2

a Sampling day 1 and 2 were 637 and 651 days post-delivery, respectively.

### Human Milk Pharmacokinetics

The total amounts in human milk on both sampling days were 0.21 µg/day and 0.37 µg/day for sildenafil and 3.18 µg/day and 6.74 µg/day for bosentan. Trough concentrations were 1.43 μg/L for sildenafil and 24.5 μg/L for bosentan. The highest concentrations in human milk were measured 4 h after dose intake for sildenafil (5.65 μg/L) and after 5 h 55 min for bosentan (86.1 μg/L). Since time between first and last of three samples at each 24 h sampling day was 12 h, AUC_0–12h_ was calculated. The average AUC_0–12h_ in human milk was 33.5 μg/L*h for sildenafil and 573 μg/L*h for bosentan. Average steady-state human milk concentrations based on AUC_0–12h_ were 2.84 μg/L for sildenafil and 49.0 μg/L for bosentan.

### Infant Exposure

The Daily Infant Dosage (DID) for the nursing infant after maternal intake of sildenafil and bosentan was calculated using Equation (1) (Table 3), with n the number of milk expression sessions within the 24 h’ time interval, and milk volume as recorded for each expression session:

$$DID\;\left(\frac{\frac{µg}{kg}}{day}\right)\;=\sum_{i=1}^{n}\left(\frac{Milk\;Concentration_{\;i}\left(\frac{µg}{L}\right)*Milk\;Volume_{i}\;\left(L\right)}{Infant\;weight\;\left(kg\right)}\right)$$ (1)

**TABLE 3:** Equations used throughout the case report.

Parameter	Equation
Daily Infant Dosage (DID) (µg/kg/day)	Equation used for the mother-infant pair: $DID\;\left(\frac{\frac{µg}{kg}}{day}\right)=\sum_{i=1}^{n}\left(\frac{Milk\;Concentration_{\;i}\left(\frac{µg}{L}\right)*Milk\;Volume_{i}\;\left(L\right)}{Infant\;weight\;\left(kg\right)}\right)$
	Equation used for an exclusively breastfed infant: $DID\;\left(\frac{\frac{µg}{kg}}{day}\right)=Average\;Steady-State\;Milk\;Concentration\left(\frac{µg}{L}\right)*Infant\;Milk\;Intake\;\left(\frac{\frac{L}{kg}}{day}\right)$
Relative Infant Dose (RID) (%)	$\mathrm{RID}\;\left(\%\right)=\frac{DID\;\left(\frac{\frac{µg}{kg}}{day}\right)}{Daily\;Maternal\;Dose\;\left(\frac{µg}{day}\right)/Maternal\;Weight\;\left(kg\right)}*100$
Relative Infant Therapeutic Dose (RID_therapeutic_) (%)	$\mathrm{RI}D_{therapeutic}\;\left(\%\right)=\frac{DID\;\left(\frac{\frac{µg}{kg}}{day}\right)}{Daily\;Therapeutic\;Infant\;Dosage\;\left(\frac{\frac{µg}{kg}}{day}\right)}*100$
Average Infant Medicine Plasma Concentration (µg/L)	$Average\;Infant\;Medicine\;Plasma\;Concentration\;\left(\frac{µg}{L}\right)=\frac{DID\;\left(\frac{\frac{µg}{kg}}{day}\right)}{Apparent\;Oral\;Clearance_{Infants}\;\left(\frac{\frac{L}{h}}{kg}\right)*24h}$

The DIDs on sampling day 1 and 2 were 0.02 and 0.03 μg/kg/day for sildenafil and 0.29 and 0.60 μg/kg/day for bosentan, respectively. It is relevant to mention that the DID strongly depends on the milk intake, which was low in this case as the infant also received complementary foods (Table 2).

The Relative Infant Dose (RID) was calculated to compare the daily dose the infant receives via breastfeeding relative to the maternal dose using Equation (2) (Table 3), with a maternal weight of 83 kg:

$$\mathrm{RID}\;\left(\%\right)=\frac{DID\;\left(\frac{\frac{µg}{kg}}{day}\right)}{Daily\;Maternal\;Dose\;\left(\frac{µg}{day}\right)/Maternal\;Weight\;\left(kg\right)}*100$$ (2)

The RIDs for this infant on both sampling days were 0.003% and 0.005% for sildenafil and 0.01% and 0.02% for bosentan, respectively.

In addition, Relative Infant Therapeutic Dose (RID_therapeutic_) was calculated using Equation (3) (Table 3), to compare the Daily Infant Dosage via breastfeeding relative to the usual Daily Therapeutic Infant Dosages for sildenafil (1,500 μg/kg/day) and bosentan (4,000 μg/kg/day) (Stayveer, 2022a; Stayveer, 2022b).

$$\mathrm{RI}D_{therapeutic}\;\left(\%\right)=\frac{DID\;\left(\frac{\frac{µg}{kg}}{day}\right)}{Daily\;Therapeutic\;Infant\;Dosage\;\left(\frac{\frac{µg}{kg}}{day}\right)}*100$$ (3)

The RID_therapeutic_ on sampling day 1 and 2 were 0.001 and 0.002% for sildenafil and 0.007 and 0.02% for bosentan, respectively.

The systemic exposure of the infant was estimated using Equation (4) (Table 3):

$$Average\;Infant\;Medicine\;Plasma\;Concentration\;\left(\frac{µg}{L}\right)=\frac{DID\;\left(\frac{\frac{µg}{kg}}{day}\right)}{Apparent\;Oral\;Clearance_{Infants}\;\left(\frac{\frac{L}{h}}{kg}\right)*24h}$$ (4)

The apparent oral clearance of sildenafil (2.04 L/h/kg) in infants (2–121 days postnatal) as previously reported based on a one-compartment PK analysis was used for our calculations according to Equation (4) (Ahsman et al., 2010). The calculated Average Infant Sildenafil Concentration in the present study was 0.0004 μg/L at day 637 and 0.0007 μg/L at day 651.

The apparent oral clearance of bosentan in infants (0.35 L/h/kg) was obtained by dividing the dose by the steady-state AUC_0–24h_ in infants (>34 weeks gestation, <7 days old) as referenced in a previous study (Steinhorn et al., 2016). This resulted in an estimated Average Infant Bosentan Concentration of 0.03 μg/L at day 637 and 0.07 μg/L at day 651 in the present study.

Finally, and because of the low human milk volume intakes in this case report, other scenarios were explored. First, the DID was also calculated based on the average steady-state milk concentration and average infant milk intake, giving an idea about the amount of exposure in an exclusively breastfed infant (Equation (5), Table 3):

$$DID\;\left(\frac{\frac{µg}{kg}}{day}\right)=Average\;Steady-State\;Milk\;Concentration\left(\frac{µg}{L}\right)*Infant\;Milk\;Intake\;\left(\frac{\frac{L}{kg}}{day}\right)$$ (5)

Based on an average infant milk intake of 0.15 L/kg/day, the DID was 0.43 μg/kg/day (RID 0.06%) for sildenafil and 7.43 μg/kg/day (RID 0.24%) for bosentan. Second, for an average infant milk intake in early infancy of 0.20 L/kg/day, the DID was 0.57 μg/kg/day (RID 0.08%) for sildenafil and 9.79 μg/kg/day (RID 0.33%) for bosentan. The calculated DIDs for an exclusively breastfed infant are substantially lower than the usual daily therapeutic infant doses for sildenafil and bosentan.

## Discussion

This case report provides human milk levels of sildenafil and bosentan along with the reported health outcomes of the nursing infant. To our knowledge, there is no published information about the levels of bosentan in human milk. A single case report previously described the milk concentrations of sildenafil (1.64–4.49 μg/L) and N-desmethylsildenafil (1.18–1.82 μg/L) in a woman receiving multiple oral doses of 20 mg sildenafil daily for PAH (Wollein et al., 2016). We presently observed similar sildenafil concentrations ranging from 1.43 μg/L to 5.65 μg/L.

Another case report described the use of sildenafil and bosentan during pregnancy and postpartum in a 23-year old woman with Eisenmenger’s syndrome (Molelekwa et al., 2005). The doses or human milk concentrations were not reported. The authors stated that the infant was nursed for 11 weeks with good outcome. Unfortunately, the infant died from a respiratory syncytial virus infection at 26 weeks post-delivery (Molelekwa et al., 2005). In our case study, the mother did not report any adverse effects, health problem or hospitalization of the infant from birth until the last sampling day. Treatment with sildenafil and bosentan was initiated more than 6 months after delivery.

The RID in our case study for both sildenafil and bosentan were well below 1%, amounting to 0.06 and 0.24% for sildenafil and bosentan, respectively. Although questions can be raised about the use of an empirical cut-off value (typically 10%), it is highly unlikely that these very low RID’s would result in toxic exposure to the infant, especially for sildenafil as there are reassuring safety data in infants (Samiee-Zafarghandy et al., 2014). This was also confirmed by comparing the calculated DID to usual doses administered to infants for therapeutic reasons. Finally, when estimating infant systemic concentrations based on reported clearance values for these medicines in infants, negligible systemic exposure in the nursing infant through breastmilk (Equation (4)) was predicted. For sildenafil, the exposure might even be lower due to the drug-drug interaction with bosentan in the infant (Sandoz nv/sa, 2020). For bosentan, this interaction might lead to an increase in exposure, although still very low, also because clearance values in infants were obtained from studies in very young infants and therefore most likely underestimated (Steinhorn et al., 2016; Accord Healthcare B.V., 2021). Other medicines taken by the mother are not expected to interfere with the concentrations of sildenafil and bosentan.

This study has several limitations. First, it includes only a single mother-infant pair. In addition, the 21-months old infant was not exclusively breastfed. Human milk volume and composition in this setting and period of lactation also differs from mature milk during exclusive breastfeeding (Casey, 1989). During the first days postpartum, the immature blood-milk barrier might result in higher concentrations of medicines in colostrum (Nguyen and Neville, 1998). At the same time, the volume of colostrum ingested by the infant is much lower compared to mature human milk. Furthermore, infants have immature pharmacokinetic and pharmacodynamic processes in the first 6 months (van den Anker et al., 2018). Both sildenafil and bosentan are metabolized by CYP enzymes to (partially) active metabolites. Genetic polymorphisms in CYP3A4 and CYP2C9 are expected to contribute to interindividual variabilities in exposure to these medicines and their metabolites (Chen et al., 2014; Tang et al., 2020). Physiologically-based pharmacokinetic modelling and simulation could be applied to predict the possible impact of such polymorphisms on pharmacokinetics of these medicines. The genotypes of the mother-infant pair for these enzymes were not known in our case report. Consequently, it is not recommended to extrapolate these data to the entire population of lactating mothers and their infants. However, as there is currently almost no data available about the use of these two medicines during breastfeeding, this case contributes to the current knowledge supporting clinicians to make evidence-based decisions in practice. Second, blood samples from the mother-infant pair for medicine quantification have not been collected. Although using a structured (sampling) approach for human milk, the number of samples collected remains limited. However, samples were collected under steady-state conditions, limiting fluctuations in concentrations. Finally, a LC-MS/MS method was developed and able to quantify bosentan and sildenafil in human milk, although their main metabolites were not determined in the present study (Supplementary Data). The contribution of N-desmethylsildenafil to the pharmacological activity is 36% compared to the parent compound (Sandoz nv/sa, 2020). N-desmethylsildenafil is highly bound to plasma proteins and is further metabolized. Similarly, the active metabolite of bosentan is expected to contribute less than 20% to the total pharmacological activity (Accord Healthcare B.V., 2021). The metabolite is excreted into the bile. Overall, based on their lower lipophilicity compared to the parent compounds, metabolites are expected to show lower transfer rates across the blood-milk barrier.

In summary, the concentrations of both sildenafil and bosentan measured in human milk collected at 21 months after delivery were very low, especially when compared to the maternal and commonly used therapeutic infant doses. In addition, in this case study no negative effect on the general health of the nursing infant was reported by the mother.

## Data Availability

The datasets for this article are not publicly available due to concerns regarding participant/patient anonymity. Requests to access the datasets should be directed to the corresponding authors.

## Footnotes

1
https://www.who.int/health-topics/breastfeeding#tab=tab_1.

2
https://www.imi-conception.eu/.
