# Supplementary material for: Case Report: Bosentan and Sildenafil Exposure in Human Milk - A Contribution From the ConcePTION Project
Source: Front Pharmacol. 2022 Jun 15;13:881084. doi: 10.3389/fphar.2022.881084 (PMC9240352; doi:10.3389/fphar.2022.881084)
Supplement: Supplementary file 2 [file DataSheet2.docx]

Supplementary Material

# Bioanalysis method description

The study samples were analyzed in accordance with the bioanalytical method which determines both sildenafil and bosentan concentrations in human breast milk using LC-MS/MS. The isotopes sildenafil-D8 and bosentan-D4 were used as internal standards (IS) correspondingly. The quantification range was 1 – 200 ng/mL for both analytes. The imprecision on the sample analysis was ≤ 6%.

The instrumental part of the method was developed on a Shimadzu UHPLC-MS/MS consisting of two LC-30AD pumps with a DGU-20A5R degassing unit, SIL-30AC autosampler, CTO-20AC column oven and CBM-20A system controller, coupled with LCMS-8050 triple quadrupole mass spectrometer using electrospray ionization (ESI) source operating at positive mode.

Chromatography separation was carried out on an Phenomenex Kinetex™ F5 (50 x 2.1 mm, 1.7µm) column at 40 °C. The autosampler temperature was set at 15°C. The mobile phase was 0.01% formic acid in water and in acetonitrile with a gradient mode. The system was equilibrated with a flow rate of 0.4 mL/min at 30% organic, and a linear gradient was used with 30% to 50% organic from 1.0 to 2.0 minutes, holding for 1.0 minute and then 50% to 30% organic for 0.5 minutes. The overall runtime of the method was 5.5 minutes. Retention time of sildenafil and its IS were at 1.25 min, bosentan and its IS at 3.25 min (Supplementary Figure 2).

Analysis was performed with an ionizing voltage of 4000 V. The interface temperature was set at 300°C and desolvation line temperature at 250°C, with ultrahigh-purity nitrogen for the drying gas (10 L/min), nebulizer gas (3 L/min) and heating gas (10 L/min). Multiple reaction monitoring (MRM) was carried out using argon as collision gas (CID), with a dwell time of 50 ms for each transition. Sildenafil was detected by monitoring the transitions at m/z 475.0 → 58.3 as a quantitative and 475.0 → 100.3 as a qualitative, with the collision energy at -39 and -28 V, correspondingly; the monitored transitions of bosentan were at m/z 552.2 → 202.2 as quantitative and m/z 552.2 → 280.1 as qualitative, with the collision energy at -35 and -39 V, correspondingly; the monitored transitions of sildenafil-D8 (IS1) were at m/z 483.2 → 62.3 as quantitative and m/z 483.2 → 108.1 as qualitative, with the collision energy at -49 and -28 V, correspondingly; the monitored transitions of bosentan-D4 (IS2) were at m/z 556.2 → 202.0 as quantitative and m/z 556.2 → 106.3 as qualitative, with the collision energy at -33 and -62 V, correspondingly.

The data were acquired and analyzed by LabSolutions software (Shimadzu; version 6.81).

Stock solutions of the analytes and ISs were individually prepared in methanol at 1 mg/mL concentration. A secondary stock solution was made for further preparation of working solutions, which contains both analytes at 10 µg/mL in methanol : water (1:1 v/v). The IS secondary stock solution was prepared alike. Preparation of the calibration curve standards (CCS) and quality control (QC) working solutions were done by diluting appropriate volumes of the secondary stock solution in methanol : water (1:1 v/v). The IS secondary stock solution was diluted in methanol to obtain an IS-Precipitation Solution. CCS and QC samples were prepared by spiking CCS and QC working solutions into blank human breast milk a ratio of 1:19.

Milk samples were thawed at room temperature, and analytes were extracted by protein precipitation using 300 µL IS-Precipitation Solution into 100 µL sample. After centrifugation under 12000 rpm for 10 minutes at 21°C, 200 µL of each sample’s supernatant was added into 200 µL water in glass vials and mixed well. 10 µL was injected into the LC–MS/MS system for analysis.

## Bioanalysis method performance

### Calibration Curve Accuracy Results

Calibration curve standards were freshly spiked. Linear regression model weighted 1/x^2^ was used which adequately described the concentration – peak area relationship. The summary of CCS’s results obtained during the study samples analysis is presented in Supplementary Table 1.

Acceptance criteria:

At least 75% of calibration curve standards, when back calculated including the ULOQ (CCS 8) must fall within 15.0 % except of LLOQ (CCS 1) where it must be within 20.0 % of the nominal value. Values falling outside these limits must not be used to calculate regression analysis. R^2^ must be no less than 0.985 for any sample analysis.

### Quality Control Accuracy and Precision Results

The accuracy of a method is defined as the proximity of the determined values to the actual concentration of the sample. The accuracy is expressed as %Accuracy. The precision of a method is defined as the closeness of the results when replicate assays of a test sample are carried out and is expressed as %CV. Freshly spiked Low, Medium and High Quality Control samples were prepared in four replicates, of which two replicates were injected before, and two after the study samples. The results are represented in Supplementary Table 2.

Acceptance Criteria:

The criteria for acceptable accuracy is that the mean value must be within ±15.0% (85.0-115.0%) of the nominal value at each level. The criteria for acceptable precision is that the percent coefficient of variance (%CV) be within 15.0% at each level.

# Supplementary Figures and Tables

## Supplementary Figures


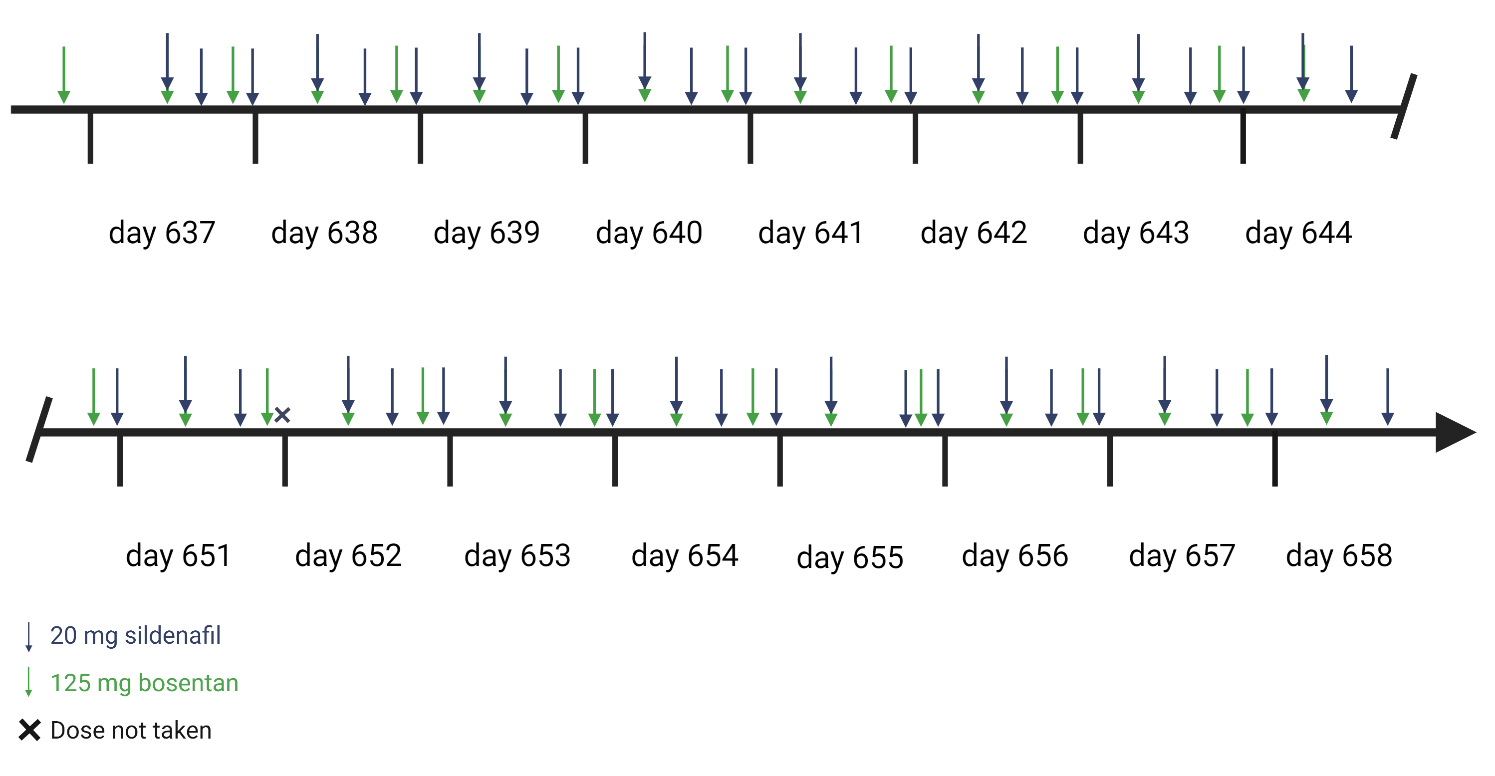


**Supplementary Figure 1.** *The figure gives an overview of the medicine history in function of the time after delivery. The mother was treated with sildenafil Balcoga film-coated tablets (20 mg, 3x/day) and Bosentan Accord film-coated tablets (125 mg, 2x/day) for pulmonary arterial hypertension. She also took Vitamine D3 Holland & Berret (oral spray, 25 µg/day) each morning and Ibuprofen (400 mg, occasionally), but this is not expected to interfere with the studied medicines.*


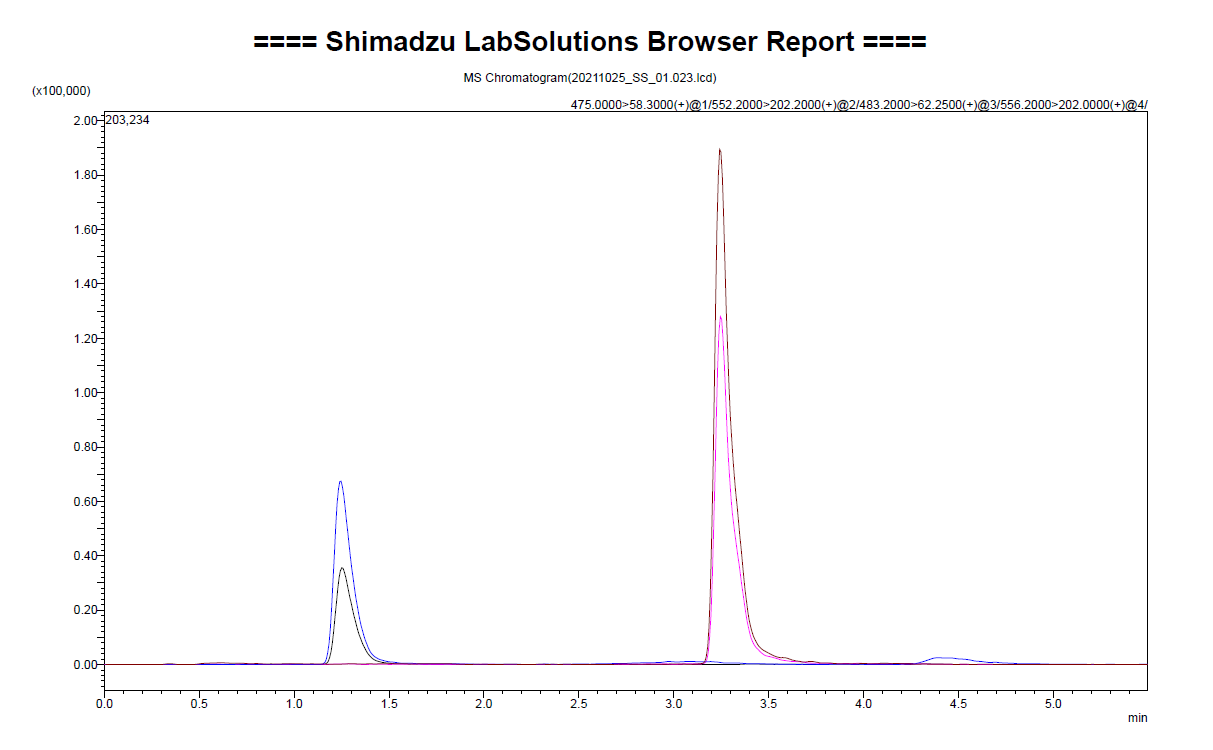


Supplementary Figure 2 *The chromatogram shows sildenafil (blue), sildenafil-D8 (black), bosentan (brown) and bosentan-D4 (pink).*

## Supplementary Tables

Supplementary Table 1 Summary of CCS’s obtained during sample analysis

| **Analyte** | | **Sildenafil** | | **Bosentan** | | |
| --- | --- | --- | --- | --- | --- | --- |
| **Calibration Curve Standard** | **Nominal Conc. (ng/mL)** | **Concentrations (ng/mL)** | **% Accuracy** | **Concentrations (ng/mL)** | **% Accuracy** | |
| **CCS 1** | **1.000** | 0.992 | 99.2 | 0.993 | 99.3 | |
| **CCS 2** | **2.000** | 2.000 | 100.0 | 2.033 | 101.7 | |
| **CCS 3** | **4.000** | 4.114 | 102.8 | 4.030 | 100.7 | |
| **CCS 4** | **10.000** | 10.315 | 103.1 | 9.872 | 98.7 | |
| **CCS 5** | **20.000** | 19.308 | 96.5 | 19.288 | 96.4 | |
| **CCS 6** | **50.000** | 48.594 | 97.2 | 49.855 | 99.7 | |
| **CCS 7** | **100.000** | 101.688 | 101.7 | 102.333 | 102.3 | |
| **CCS 8** | **200.000** | 198.797 | 99.4 | 202.256 | 101.1 | |
| **slope** | | 0.0338963 | | 0.0460739 | | |
| **intercept** | | +0.00811315 | | -0.0000498197 | |  |
| **R^2^** | | 0.999 | | 1.000 | | |

Supplementary Table 2 QC’s results for the study batch

| **Analyte** | **Sildenafil** | | | **Bosentan** | | |
| --- | --- | --- | --- | --- | --- | --- |
| **QC Level** | LQC  (3 ng/mL) | MQC  (15 ng/mL) | HQC  (150 ng/mL) | LQC  (3 ng/mL) | MQC  (15 ng/mL) | HQC  (150 ng/mL) |
| **Concentration (ng/mL)** | 2.988 | 15.295 | 152.471 | 3.025 | 15.614 | 153.094 |
|  | 2.980 | 15.395 | 143.698 | 3.089 | 15.005 | 143.502 |
|  | 3.096 | 15.065 | 152.489 | 2.955 | 14.219 | 145.562 |
|  | 3.089 | 15.215 | 157.932 | 2.971 | 14.312 | 146.511 |
| **Average** | 3.038 | 15.243 | 151.648 | 3.010 | 14.788 | 147.167 |
| **CV (%)** | 2.1 | 0.9 | 3.9 | 2.0 | 4.4 | 2.8 |
| **Accuracy (%)** | 101.3 | 101.6 | 101.1 | 100.3 | 98.6 | 98.1 |
